# Patients’ and physicians’ gender and perspective on shared decision-making: A cross-sectional study from Dubai

**DOI:** 10.1371/journal.pone.0270700

**Published:** 2022-09-01

**Authors:** Mohamad Alameddine, Farah Otaki, Karen Bou-Karroum, Leon Du Preez, Pietie Loubser, Reem AlGurg, Alawi Alsheikh-Ali

**Affiliations:** 1 Department of Clinical Nutrition and Dietetics, College of Health Sciences, Research Institute of Medical & Health Sciences (RIMHS), University of Sharjah, Sharjah, United Arab Emirates; 2 Strategy and Institutional Excellence, Mohammed Bin Rashid University of Medicine and Health Sciences, Dubai, United Arab Emirates; 3 Department of Health Management and Policy, Faculty of Health Sciences, American University of Beirut, Beirut, Lebanon; 4 Cardiac Anesthesiology, The City Hospital, Dubai Health Care City, Dubai, United Arab Emirates; 5 Mediclinic Middle East, Dubai, United Arab Emirates; 6 College of Medicine, Mohammed Bin Rashid University of Medicine and Health Sciences, Dubai, United Arab Emirates; University of Pennsylvania, UNITED STATES

## Abstract

**Background:**

Delivering patient-centered care is a declared objective of many health delivery systems globally, especially in an era of value-based health care. It entails the active engagement of the patients in healthcare decisions related to their health, also known as shared decision making (SDM). Little is known about the role of gender in shaping the perspective of patients on their opportunity for engaging in SDM in the Arabian Gulf Region. The aim of this study is to investigate the role of gender in shaping patients’ perspectives toward their opportunity for SDM in Dubai, UAE.

**Methods:**

This study utilized a cross-sectional survey consisting of sociodemographic questions and the 9-item Shared Decision-Making Questionnaire (SDM-Q-9). A total of 50 physicians (25 females and 25 males), practicing at a large private healthcare delivery network in Dubai, were recruited using convenience sampling. Ten patients of every recruited physician (5 male and 5 female) were surveyed (i.e., a total of 500 patients). Statistical analysis assessed the differences in patients’ perceptions of physician SDM attitude scores by physicians’ and patients’ gender using independent t-test, ANOVA-test, and Chi-square analyses.

**Findings:**

A total of 50 physicians and 500 patients (250 male patients and 250 female patients) participated in this study. The odd of patients agreement was significantly lower for male physicians, compared to their female counterparts, on the following elements of SDM: the doctor precisely explaining the advantages and disadvantages of the treatment (OR = 0.55, 95%CI: 0.34–0.88, p = 0.012); the doctor helping them understand the information (OR = 0.47, 95%CI: 0.23–0.97, p = 0.038), the doctor asking about preferred treatment option (OR = 0.52, 95%CI: 0.35–0.77, p = 0.001), and the doctor thoroughly weighting the different treatment options (OR = 0.60, 95%CI: 0.41–0.90, p = 0.013). No significant associations were observed between patients’ gender and their perception of their opportunity for SDM. Likewise, no significant associations were observed between the same or different physician-patient gender and patients’ perception of physicians’ SDM attitudes. Statistically significant associations were observed between physician-patient gender and preferred treatment option for patients (p = 0.012).

**Conclusion:**

Study findings suggest that while there were no differences in patients’ perspective on SDM by the gender of patients, significant differences were observed by the gender of physicians. Female physicians, compared to their male counterparts, were more engaged in SDM, with both male and female patients. Male physician-female patient dyad received the lowest scores on SDM. This could be explained by the cultural, social, and religious sensitivities that infiltrate the physician-patient relationship in the Arab contexts. Despite the multi-cultural nature of the country, some female patients may still experience some discomfort in opening up and in discussion preferences with male physicians. For physicians, striking the right balance between assertiveness and SDM is necessary within the cultural context, especially among male providers. Offering targeted learning and development programs on the importance and practice of SDM is also necessary to ensure equitable opportunity for engagement in SDM for all patients irrespective of the gender of their provider.

## Background

During the past few decades, healthcare organizations witnessed a growing shift from the traditional paternalistic model of decision making in clinical settings towards a patient-centered care model [1]. This is especially true for value-based health care [2], where the main aspect of patient-centered care entails active engagement of the patients in their own healthcare decision making process, which is also known as shared decision making (SDM) [1, 3]. SDM constitutes three domains: information-sharing between patients and providers, deliberation about the advantages and disadvantages of treatment options, and decision-making about a treatment plan that is approved by both the patient and the physician [4, 5]. In this model of patient-provider communication, both patients and providers bring their own experiences, health literacy, and identities to the encounter, with variable levels of discordance [4].

Implementing SDM in clinical practice has been associated with improved patients’ self-reported outcomes and their understanding of risks, as well as greater satisfaction with the consultation process [6–8]. In addition, SDM improves quality of care and patient adherence to medication, and consequently contributes to the optimization of health costs [7]. As such, the recent surge in emphasis on value‐based health care is expected to be coupled with increased attention to SDM, especially when using patient‐reported outcome measures [9, 10]. The employment of SDM requires major commitment from healthcare professionals with robust communication skills [11–13]. In other words, physicians with active listening skills are fundamental assets to establish SDM, and consequently, to encourage patients to share information about their personal life style, living situation, and personal preferences of treatment [1]. A study by Zisman-Ilani et. al, revealed several issues related to the patient-physician relationship such as lack of time and training, the role of insurance companies, the incentive structure, and lack of support for physicians [14]. Another study investigating the barriers to communication between family physicians and patients in Dubai showed that female patients experience more time limitations during consultation where not all their issues are addressed as compared to their male counterparts [15]. That said, gender is known as an important variable for communication styles, decision-making styles, values, and preferences [7]. As such, the concordance or discordance between the gender of the physician and the patient was found to play a key role in the interaction and communication between physicians and patients [16, 17].

Literature reports differences between gender dyads and SDM interactions. Female physician-female patient dyad is the most patient-centered, characterized by more psychosocial talk (e.g., lifestyle, and social aspects), bio-medical talk (e.g., therapeutic and medical concerns), and the longest consultations. In contrast, female physician-male patient interactions are characterized by less ease, more tension around gender role conflict, and unfriendly voice tones [18, 19]. Gender concordance may also improve overall patient-physician interaction by encouraging patients’ trust, and enhancing communication and patient satisfaction [20]. Studies showed that gender concordance may be linked to improved hypertension and diabetes outcomes as well as to the delivery of preventative counseling [20]. Indeed, better patient-physician communication have a positive effect on the overall patient experience, and consequently, improved health outcomes [20]. Available data on cross-gender encounters reveal that female physicians display a more patient-centered attitude [21]. As a matter of fact, female physicians were found to give more information and emotional support, talk more encouragingly, and put greater effort into partnership building [22]. Moreover, patients of female physicians, both male and female, were more engaged in discussions with their physician, expressed themselves more freely, shared more information, and disclosed partnership statements [23].

## Local context

The current investigation takes place in Dubai, a populous commercial and touristic hub on the Eastern side of the Arabian Peninsula. Dubai is a multi-cultural cosmopolis with a population of 3.5 million, and a large expatriate population coming from more than 200 countries around the globe [24]. Dubai’s healthcare sector has developed remarkably in the past few years, providing exceptional opportunities for both investors and patients. The healthcare sector in Dubai is divided between public and private providers, which are primarily regulated by the Dubai Health Authority (DHA). It is characterized by a high-tech medical infrastructure serving not only the local market, but also wider regional demand [25].

Participants were recruited from Mediclinic Middle East, a large private healthcare delivery network in the UAE. Mediclinic Middle East offers several health services for both genders in the UAE, and treats priority issues including hypertension, diabetes, obesity, and cardiovascular diseases [26, 27].Within Dubai, the network is comprised of 3 hospitals and 10 independent clinics.

Dubai’s culture is rooted in Islamic and Arabic traditions, one might expect that patients in the city may feel more comfortable in interactions with physicians of their own gender [28]. Furthermore, according to Islamic doctrine, females are expected to seek care from a same gender clinician, especially in gender sensitive specialties (e.g. OBSGYN), unless there is no female clinician available to offer services to other females [29, 30]. To date, rarely has gender been examined as a potential factor influencing the extent of SDM during consultations in the Arab Region. Yet, the SDM literature lacks studies that represent different cultures from countries outside the US, Canada, EU, and Australia [31]. To the best knowledge of the authors, the current investigation is the first of its kind in Dubai. As such, the current study aims at investigating the role of gender in shaping the perspectives of patients on their opportunity to engage with their physician in SDM. Study findings can be leveraged to help in shaping a better patient experience and in ensuring equitable delivery of healthcare in Dubai and other similar contexts.

## Methodology

### Research design

This study employs a non-experimental cross-sectional survey design to capture quantitative data regarding the perception of patients and physicians in Dubai in relation to SDM. The study leverages this data to investigate potential associations in relation to the effect of gender on the perspective of patients of the opportunity of engaging in SDM.

### Instruments

The study collected data from both patients and physicians using two questionnaires. The questionnaire for patients was composed of two segments. The first segment included questions collecting basic socio-demographic data, including: age, gender, educational level, and nationality of patients. In the second segment, patients were asked to complete the 9-item SDM Questionnaire for patients (SDM-Q-9).

A second survey questionnaire collected similar data from physicians and included two sections. The first collected personal and professional information about the physician (age, gender, specialty, nationality, years of experience, and type of practice institution). The second section was composed of the SDM-Q-9 questionnaire, adjusted to capture the perspective of physicians on their provision of the opportunity for SDM to their patients.

The response for each question in the SDM questionnaire ranged from 1 (Strongly Disagree) to 5 (Strongly Agree). The 5-point Likert scale has been used in previous studies employing the SDM-Q9 scale [32–34]. Additionally, as stated in the literature, Likert scales are simple to construct, are likely to produce high reliable scales, and are easy to read and complete [35]. Thus, for the nine questions, this instrument yields a score ranging from 9 (indicative of a very poor perception of SDM), to 45 (indicative of a very strong perception of SDM) [36]. As suggested, we rescaled the total SDM scores to a 0–100 range with higher values indicating higher SDM [36]. The questionnaire used in this study have been demonstrated to have good psychometric properties. For example, the reliability analysis of the SDM-Q-9 scale showed a Cronbach’s *α* of 0.943 [36]. The questionnaire was reviewed by an expert panel including a clinician, statistician, health services researcher, nurse, and a patient advocate to enhance the content validity. The questionnaires of both patients and physicians were available in Arabic and English. They were translated to Arabic and back translated to English by two different certified translators. In addition to the questionnaires, a clinic information sheet, gathering basic information about the physician and the clinic, was filled by the data collectors.

### Sampling strategy

To be able to detect a difference of 5 units between two groups (effect size of 0.3) [37], with a power of 90%, and a type I error of 5%, the needed sample size is 470 patients. Accordingly, the research team recruited 500 patient participants. This sample allows running a factorial analysis of variance with enough power (80%) to detect a small effect size (0.2) at the main effect with interactions included in the model.

**Fig 1:**
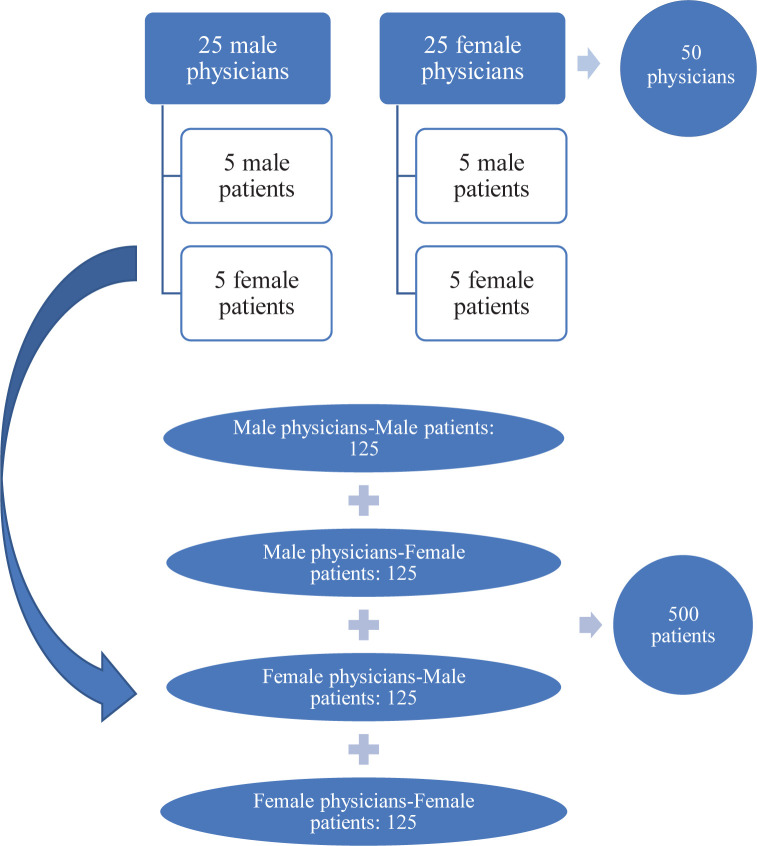
Recruitment plan for this study.

To obtain the 500 patient participants, a total of 50 physicians were recruited in the study (25 male physicians and 25 female physicians). Both groups of stakeholders were recruited using convenience sampling (Fig 1).

### Inclusion/Exclusion criteria

All physicians were included in the study except those who provide care for patients of a single gender (e.g., obstetrics and gynecology, and urology). Moreover, specialties that were likely to be dealing with critical cases were excluded: Emergency Medicine, Intensive Care Unit, Anesthesiology, and Psychiatry. Pediatricians were also excluded since the principles of SDM differ when dealing with children [38]. Patients were included in the study if they were adult aged 18+, understand Arabic or English, and are either new patients or returning patients with a new complaint (to ensure the occurrence of decision-making during the visit).

### Data collection

Convenience sampling, in two sequential recruitment phases, was utilized to achieve the desired sample size. The first phase of recruitment started with obtaining a complete list of all licensed physicians working in the target facilities. All the eligible physicians (around 200) were approached initially through the Chief Medical Officer monthly meeting in each of the included units. During the meeting, the project coordinator introduced the project and addressed any concerns. Following that, personalized emails were sent out to all the eligible physicians. The first 50 physicians (25 male and 25 female) who expressed interest to participate, and in turn submitted signed consent forms, were included in the study.

The second phase of the sampling included reaching out directly to patients of the 50 participating physicians. A total of 10 data collectors underwent a thorough training to equip them with the knowledge and skills needed to carry out data collection as per the approved research protocol of this study. They collected data from the clinics of participating physicians during the working days and hours of the physician, with data collection alternating between the morning and afternoon/evening shifts. No more than five patients were surveyed in a single visit.

The data collectors approached the individual patients after completing their consultations and prior to leaving the out-patient clinic. The patients agreeing to participate in the study were asked to provide written consents prior to completing the study questionnaire. The patients completed the questionnaires in a quiet and private space. The first ten patients (5 male and 5 female patients) who consented to participating in this study were selected. The data collectors approached a total of 714 patients out of whom 500 agreed to participate. Patients who did not agree to participate were either not in the mood to evaluate their experience, did not feel (physiologically and/ or psychologically) well enough to do so, or did not have the time to complete the questionnaire.

### Pilot study

Pilot testing was conducted on two randomly selected physicians and four patients (two for each physician). No changes were necessary to the survey questionnaire as a result of this pilot test.

### Data analysis

Collected data were coded and analyzed using IBM SPSS statistics software version 25. Descriptive analysis was carried out to summarize patient characteristics such as socio-demographics and medical conditions. Differences in SDM scores of patients by physicians’ or patients’ gender were conducted using independent t-test and ANOVA-test. Proportion of patients who strongly agreed/agreed with each statement in the SDM scale was compared between female and male patients using Chi-square test. A p-value of 0.05 was used to detect significance in all analyses used in the present study.

### Ethical approval and considerations

This study was approved by four Institutional Review Boards: Partners Human Research Committee, Mohammad Bin Rashid University of Medicine and Health Sciences, Dubai Healthcare City Authority-Regulatory Ethics Review Committee, and Dubai Health Authority-Dubai Scientific Research Ethics Committee. The participation of both patients and physicians was entirely voluntary, and written consent forms were obtained prior to any data collection initiative. Participants had the right to refuse participation, to withdraw at any point in time, and to refrain from answering any question(s). There were no incentives for participation or penalties for non-participation. The data collected was secured in a locked cabinet and in turn on a password protected computer. Anonymity and confidentiality of the respondents was guaranteed, where no personal identifiers were recorded at any stage of the study.

## Results

A total number of 50 physicians and 500 patients participated in this study. The demographic characteristics of respondents are described in Table 1. The mean (±SD) age of patients was 44.5 (±14.4), equally divided by gender (by virtue of the research design). Most of the patients (84%) had attained at least an undergraduate university education level. Physicians had an average age of 52.0 (±14.3). Most physicians were practicing at hospital-based clinics (82%), and had an average of 20.6 (±13.8) years of experience. The mean SDM-Q-9 score for the patients in this sample was 83.46 (±12.82) and that for the physicians was 88.71 (±12.07).

**Table 1:** Demographic characteristics of study sample.

Patients	N = 500
**Age**	44.51 ±14.42
Female	43.52 ±14.53
Male	45.51 ±14.25
**Gender**	
Female	250(50.0)
Male	250(50.0)
**Education level**	
≤ High school	78(16.0)
University degree	286(58.8)
Higher education	122(25.1)
**Physicians**	**N = 50**
**Age**	52.02 ±14.27
Female	48.75 ±16.00
Male	55.28 ±11.73
**Gender**	
Female	25(50.0)
Male	25(50.0)
**Years of experience**	20.62 ±13.75
**Clinic type**	
Hospital-based	41(82.0)
Polyclinic	9(18.0)

Using simple logistic regression, Table 2 displays the association between the gender of physicians and the perspective of patients on their opportunity to engage with SDM. Compared to patients cared for by female physicians, patients cared for by male physicians were less likely to strongly agree/agree that the doctor precisely explained the advantages and disadvantages of the treatment (78.3 vs. 86.6%, OR = 0.55, 95%CI: 0.34–0.88, p = 0.012). Similarly, patients of male physicians were less likely to strongly agree/agree that the doctor helped them understand the information (90.4 vs 95.2%, OR = 0.47, 95%CI: 0.23–0.97, p = 0.038), asked them about their preferred treatment option (63.7 vs. 77.1%, OR = 0.52, 95%CI: 0.35–0.77, p = 0.001), and thoroughly weighted the different treatment options (67.7 vs. 77.5%, OR = 0.60, 95%CI: 0.41–0.90, p = 0.013).

**Table 2:** Associations between the gender of physicians and the perspective of patients on their opportunity to engage with SDM.

Patients’ score	Physicians’ gender			p-value	Simple logistic regression
	Total (n = 500)/N(%)	Females (n = 250)/ N(%)	Males (n = 250)/ N(%)		OR (95%CI)
**My doctor made clear that a decision needs to be made.**					
Strongly Agree or Agree	460(92.0)	230(92.0)	230(92.0)	1.000	-
**My doctor wanted to know exactly how I want to be involved in making the decision**.					
Strongly Agree or Agree	419(84.0)	211(84.7)	208(83.2)	0.639	-
**My doctor told me that there are different options for treating my medical condition**.					
Strongly Agree or Agree	362(72.8)	187(75.4)	175(70.3)	0.199	-
**My doctor precisely explained the advantages and disadvantages of the treatment options**.					
Strongly Agree or Agree	412(82.6)	217(86.6)	195(78.3)	**0.012**	**0.55 (0.34,0.88)**
**My doctor helped me understand all the information.**					
Strongly Agree or Agree	464(92.8)	238(95.2)	226(90.4)	**0.038**	**0.47 (0.23,0.97)**
**My doctor asked me which treatment option I prefer.**					
Strongly Agree or Agree	350(70.4)	192(77.1)	158(63.7)	**0.001**	**0.52 (0.35,0.77)**
**My doctor and I thoroughly weighed the different treatment options.**					
Strongly Agree or Agree	362(72.5)	193(77.5)	169(67.6)	**0.013**	**0.60 (0.41,0.90)**
**My doctor asked me which treatment option I prefer.**					
Strongly Agree or Agree	391(78.4)	203(81.2)	188(75.5)	0.122	-
**My doctor and I thoroughly weighed the different treatment options.**					
Strongly Agree or Agree	447(89.6)	229(92.0)	218(87.2)	0.081	-
**Overall, I was happy with my interaction with the physician**					
Strongly Agree or Agree	466(93.2)	234(93.6)	232(92.8)	0.722	-

No significant associations were observed between patients’ gender and their perception on their opportunity to engage in SDM with their physician. Likewise, no significant associations were observed between same or different physician-patient gender and their perception on the opportunity to engage in SDM with their physician (S1 Table).

Table 3 reveals that the only significant associations across the different physician-patient gender dyads and the perspective of patients on their opportunity to engage with SDM was observed on the doctor discussing the preferred treatment option with their patient (p = 0.012). Male patients of female physicians perceived the highest levels related to this dimension of SDM, and the female patients of male physicians had relatively lowest perception on this dimension of SDM.

**Table 3:** Associations of physician-patient gender and the perspective of patients on their opportunity to engage with SDM.

SDM attitudes of patients	MM (N = 125)/ N(%)	MF (N = 125)/ N(%)	FM (N = 125)/ N(%)	FF (N = 125)/ N(%)	p-value
**My doctor made clear that a decision needs to be made.**					
Strongly Agree or Agree	115(92.0)	115(92.0)	113(90.4)	117(93.6)	0.833
**My doctor wanted to know exactly how I want to be involved in making the decision**.					
Strongly Agree or Agree	101(80.8)	107(85.6)	103(82.4)	108(87.1)	0.511
**My doctor told me that there are different options for treating my medical condition**.					
Strongly Agree or Agree	83(66.4)	92(74.2)	90(73.2)	97(77.6)	0.243
**My doctor precisely explained the advantages and disadvantages of the treatment options**.					
Strongly Agree or Agree	97(77.6)	98(79.0)	107(85.6)	110(88.0)	0.087
**My doctor helped me understand all the information.**					
Strongly Agree or Agree	113(90.4)	113(90.4)	120(96.0)	118(94.4)	0.208
**My doctor asked me which treatment option I prefer.**					
Strongly Agree or Agree	**80(64.5)**	**78(62.9)**	**98(78.4)**	**94(75.8)**	**0.012**
**My doctor and I thoroughly weighed the different treatment options.**					
Strongly Agree or Agree	85(68.0)	84(67.2)	96(77.4)	97(77.6)	0.104
**My doctor asked me which treatment option I prefer.**					
Strongly Agree or Agree	93(74.4)	95(76.6)	103(82.4)	100(80.0)	0.427
**My doctor and I thoroughly weighed the different treatment options.**					
Strongly Agree or Agree	108(86.4)	110(88.0)	113(91.1)	116(92.8)	0.335
**Overall, I was happy with my interaction with the physician**					
Strongly Agree or Agree	119(95.2)	113(90.4)	118(94.4)	116(92.8)	0.449

## Discussion

This was the first study in the UAE to assess the effect of the gender of patients and physicians on the perspective of patients on their opportunity to engage in SDM. Findings of this study revealed an overall high rating of opportunity for SDM by all patients and physicians. It, however, also suggested that the areas where SDM is better exercised by female physicians include: supporting patients in understanding technical information, explaining the advantages and disadvantages of the treatment options, and thoroughly weighing them, and jointly selecting the preferred treatment option. In this study, no significant association was found between same or different physician-patient gender dyads and the perspective of patients on their opportunity to engage in SDM. Therefore, the concordance/discordance of gender between patients and physicians did not seem to make a difference in patients’ perceived opportunity for SDM.

Since the days Abraham Flexner prepared his seminal report on the future of medical education in 1910, SDM has been at the forefront of discussions. Flexner emphasized on the synergy between scientism and humanism, urging all medical schools to teach the emotional as well as the scientific [39]. More than a century later, the medical and scientific communities are still striving to strike the right balance to ensure equitable and standardized care to patients while controlling for the inevitable differences in the characteristics of the patients and their providers. Understanding the variables that influence the opportunity that physicians extend to their patient to participate in SDM is of pivotal importance. Such variables include physician-specific characteristics, patient-related antecedents, the environment, and the interpersonal dynamic between the physicians and their patients [40]. The gender of the physician is indeed a significant determinant of SDM since it influences clinical practice, and thus, the patient-physician relationship [41].

The current study’s findings which highlight that the patients of female physicians tend to perceive a better opportunity for SDM, is in synch with previous investigation. Similar to this study, female physicians were reported to provide more subjective and objective information, engage more in psychosocial counseling, and involve patients more in decision making [18]. In contrast, male physicians were relatively more focused on the technical aspects of the clinical consultation (e.g., physical examination), are more assertive, and give more advice [18]. Studies also showed that female physicians spend more time with patients, relative to male physicians, and that patients are more satisfied with their communication and interaction with female physicians [42]. It is worth noting that physicians in our study, work within the same healthcare system, consequently there is no difference in the time allocated to patients by the gender of physicians.

Furthermore, it has been suggested that females, as a group, are more likely to respond to unspoken demands and implicit discomfort than males [43]. A recent study among diabetic patients, showed that patients assume that female physicians would be more empathetic and caring, whereas male physicians are usually more reluctant to discuss personal matters [44]. Therefore, it was concluded that patients have different expectations of care and SDM approach based on their physician’s gender. Knowing that physicians in this study were working within the same environment in terms of organizational values, incentives, and design, it seems that gendered social norms were also pervasive. Having said that, it must be noted that cultural, social, and religious sensitivities still infiltrate the physician-patient relationship in the Arab contexts [28, 29]. A study from Saudi Arabia, Bahrain, and the UAE showed that employee’s gender has a significant interaction effect on customer’s comfort, feedback willingness, and satisfaction with service encounter [28]. Female physicians are expected to motivate the patients and assist in finding external peer support programs, whereas male physicians are expected to only deliver services related to their professional roles such as referrals and prescriptions [44]. Future studies could investigate if there is an interaction effect of gender and communicative behavior of physicians on patients’ perceived opportunity for SDM. Perhaps the communication style of physicians has a stronger impact than gender itself [45, 46].

Interestingly, in this study, female physician-male patient dyad received the highest SDM scores on treatment options, followed by female physician-female patient. Knowing that treatment options is at the core of patient-centered care, this further indicates that female physicians are more likely to be involved in the SDM process. It seems that female physicians are successfully collaborating and engaging with male patients to remove the tension surrounding having a female in the dominant professional role. In contrast, the male physician-female patient dyad received the lowest scores on SDM. This could be attributed to factors related to patients and physicians. On the patients’ front, and despite the multi-cultural nature of the UAE, the country’s culture remains rooted in Islamic and Arabic traditions, this suggests that some female patients may feel more comfortable in opening up and interacting with female physicians. On the physicians’ front, striking the right balance between assertiveness and SDM is necessary within the cultural context, especially among male providers [28–30]. Future studies should systematically investigate the above mentioned observations, and suggest contextualized evidence-based policy and practice recommendations.

While this finding is not a novel discovery as it has been supported by previous studies [18, 42], establishing this gender pattern in an Arab-Islamic context is quite novel. This could also be explained by the efforts of the UAE government towards elimination of discrimination against women, gender equality, and supporting women who are playing a role in the development of economy [47]. According to the World Economic Forum’s Global Gender Gap Report 2017, the UAE is at the forefront of gender equality [48]. The finding could also be explained by the high educational level of the patient population in this study which may have shaped their acceptance of gender roles in a professional setting [49].

The findings of this study need to be translated and celebrated to help break any existing cultural biases related to the gender of the healthcare professional. While all physicians in this study are found to provide a good level of SDM to their patients, female physicians were better rated on several SDM attributes. On that front, the findings challenge two cultural assumptions. First, the performance of female physicians is not inferior to their male counterpart, but rather superior when it gets to patients’ evaluation of their opportunity to engage in SDM. Second, in specialties catering for both genders, the concordance of the gender of patients and providers does not ensure a higher satisfaction with care. In contrast, the male patients of female physicians had the highest score on the treatment options attribute of SDM. Having said that, the findings of this study do not by any means suggest that the care of male physicians is inferior to that of their female counterparts, it rather suggests that some male physicians could benefit from learning how to enhance their practice of SDM from their female colleagues as part of developing gender-sensitive and high-quality medical services. This is expected to translate into better outcomes of care which will directly feed into the attainment of value-based health care [9, 10]. Raising awareness on gender behavior patterns and implementing training sessions in communication and SDM skills among physicians are essential, particularly for male physicians [19]. Medical education must continue to teach compassionate and patient-centered care [50, 51]. Furthermore, future qualitative research is needed to develop a deeper understanding of what leads a patient to label a decision as shared.

### Limitations

Several shortcomings were noted in this study. First, the external validity of the findings may be limited to patient care systems that are similar to the large private provider network where the data collection took place. Yet, it must be noted that private provider networks, like the one included in this study, provide a substantial proportion of care in Dubai and the UAE. Second, although the selected network serves a diverse population, patients are usually white-collar professionals (and their families) working in Dubai and may not be representative of low-income populations or those who are underprivileged. As such, future studies should include a sample from governmental and public clinics to be more representative of the whole population. Third, this study only included a subjective assessment of patients’ perspectives regarding physicians’ SDM attitudes. It would be worthwhile for future studies to include an objective assessment of the SDM attitudes of the patients (e.g., the time spent by each physician with a patient). Fourth, as data was self-reported, it is possible that patients might have overemphasized their positive views of SDM. However, the research team tried to minimize social desirability bias through assuring the confidentiality of the collected data. Fifth, although there was no indication of selection bias during data collection and the selection of physicians was most often related to their availability, this possibility cannot be ruled out. Finally, the specialization of the male and female physicians participating in this study could not be matched which could introduce a bias due to the innate differences in practicing SDM by specialty. Future studies should include exploratory qualitative analysis, such as data from focus group sessions with random selection of patients, to develop a more thorough understanding of the reasons underlying the findings generated by this study.

## Conclusion

Gender is one of the various factors affecting the physician-patient relationship. Findings of this study suggest that while there were no differences in patients’ perspective on SDM by the gender of patients, significant differences were observed by the gender of physicians. Patients of female physicians perceived more engagement in SDM as compared to male physicians. Results also showed that the concordance/discordance of gender between patients and physicians did not seem to make a difference in patients’ perceived opportunity for SDM, suggesting that gender dyads may not act as barriers to SDM in the current study’s context. These results call for appropriate strategies to improve SDM skills among physicians, particularly male physicians, as well as raise awareness on gendered behavior patterns to ensure high quality and gender-sensitive healthcare. All this will inevitably lead to better patient outcomes of care.

## Supporting information

S1 Table Output of the inferential analysis. (DOCX)
