# Supplementary material for: Patients’ and physicians’ gender and perspective on shared decision-making: A cross-sectional study from Dubai
Source: PLoS One. 2022 Sep 1;17(9):e0270700. doi: 10.1371/journal.pone.0270700 (PMC9436052; doi:10.1371/journal.pone.0270700)
Supplement: S1 Table — (DOCX) [file pone.0270700.s001.docx]

| **SDM attitudes of patients** | **Same gender (MM or FF)**  **(n=250)** | **Different gender**  **(MF or FM)**  **(n=250)** | **p-value** |
| --- | --- | --- | --- |
| **My doctor made clear that a decision needs to be made.** |  |  |  |
| Strongly Agree or Agree | 233(92.8) | 228(91.2) | 0.510 |
| Other | 18(7.2) | 22(8.8) |  |
| **My doctor wanted to know exactly how I want to be involved in making the decision**. |  |  |  |
| Strongly Agree or Agree | 209(83.9) | 210(84.0) | 0.984 |
| Other | 40(16.1) | 40(16.0) |  |
| **My doctor told me that there are different options for treating my medical condition**. |  |  |  |
| Strongly Agree or Agree | 180(72.0) | 182(73.7) | 0.673 |
| Other | 70(28.0) | 65(26.3) |  |
| **My doctor precisely explained the advantages and disadvantages of the treatment options**. |  |  |  |
| Strongly Agree or Agree | 207(82.8) | 205(82.3) | 0.890 |
| Other | 43(17.2) | 44(17.7) |  |
| **My doctor helped me understand all the information.** |  |  |  |
| Strongly Agree or Agree | 231(92.4) | 233(93.2) | 0.729 |
| Other | 19(7.6) | 17(6.8) |  |
| **My doctor  asked me which treatment option I prefer.** |  |  |  |
| Strongly Agree or Agree | 174(70.2) | 176(70.7) | 0.899 |
| Other | 74(29.8) | 73(29.3) |  |
| **My doctor and I thoroughly weighed the different treatment options.** |  |  |  |
| Strongly Agree or Agree | 182(72.8) | 180(72.3) | 0.898 |
| Other | 69(27.2) | 69(27.7) |  |
| **My doctor  asked me which treatment option I prefer.** |  |  |  |
| Strongly Agree or Agree | 193(77.2) | 198(79.5) | 0.530 |
| Other | 57(22.8) | 51(20.5) |  |
| **My doctor and I thoroughly weighed the different treatment options.** |  |  |  |
| Strongly Agree or Agree | 224(89.6) | 223(89.6) | 0.988 |
| Other | 26(10.4) | 26(10.4) |  |
| **Overall, I was happy with my interaction with the physician** |  |  |  |
| Strongly Agree or Agree | 235(94.0) | 231(92.4) | 0.477 |
| Other | 15(6.0) | 19(7.6) |  |
